# Integrated Multi-Omics Analysis Identifies SRI as a Critical Target Promoting Gastric Cancer Progression and Associated with Poor Prognosis

**DOI:** 10.3390/cancers17213483

**Published:** 2025-10-29

**Authors:** Zhijie Gong, Weiwei Wang, Yinghao He, Jun Zhou, Qiangbang Yang, Aiwen Feng, Zudong Huang, Jian Pan, Yingze Li, Xiaolu Yuan, Minghui Ma

**Affiliations:** 1 The First School of Clinical Medicine, Southern Medical University, Guangzhou 510515, China; 2 Second Department of Gastrointestinal Surgery, Maoming People’s Hospital, Maoming Clinical Medical College, Guangdong Medical University, Maoming 525000, China

**Keywords:** gastric cancer, multi-omics analysis, prognosis, SRI, proliferation and invasion

## Abstract

**Simple Summary:**

In this study, we integrated single-cell, spatial and bulk transcriptomic analyses with experiments to uncover drivers of gastric cancer progression. We identified a malignant epithelial subset C5 and derived a five-gene prognostic signature (ASCL2, REPIN1, CXCL3, TMEM176A and SRI). This signature stratified patients into high- and low-risk groups with distinct survival, immune infiltration and therapy response profiles. Spatial transcriptomics localized TMEM176A to fibroblasts and SRI to tumor epithelium, and in vitro SRI knockdown inhibited tumor cell growth, migration and invasion. SRI represents a promising therapeutic target in gastric cancer.

**Abstract:**

**Background:** We aimed to identify key molecular drivers of gastric cancer progression and poor prognosis by integrating multi-omics analyses with experimental validation. **Methods:** Single-cell RNA-seq data were clustered to delineate major cell types. InferCNV identified tumor epithelial cells, and reclustering revealed a malignant subset with poor prognosis. The overlap between subset markers and The Cancer Genome Atlas Stomach Adenocarcinoma (TCGA-STAD) upregulated differentially expressed genes (DEGs) was modeled with univariate, LASSO-, and multivariate Cox to derive a prognostic signature. Patients were stratified according to signature scores, and group differences in survival and immunologic features were compared. Spatial transcriptomics defined the localization patterns of key signature genes. In vitro functional assays (CCK-8, colony formation, EdU incorporation, flow cytometry, Transwell migration and invasion, and wound healing) confirmed the pivotal role of SRI. **Results:** Reclustering of tumor epithelial cells yielded seven subsets (C0–C6), with C5 displaying marked malignant features and correlating with poor prognosis in multiple cohorts. Intersecting 208 genes yielded a five-gene signature (ASCL2, REPIN1, CXCL3, TMEM176A, SRI). The signature stratified patients into high- and low-risk groups. The high-risk cohort exhibited significantly poorer survival, distinct immune-infiltration patterns, elevated immune-evasion scores, and a reduced predicted response to immunotherapy. Single-cell and spatial transcriptomics localized TMEM176A to fibroblasts and SRI to the tumor epithelium. Finally, in vitro knockdown of SRI inhibited tumor cell proliferation, migration and invasion. **Conclusions:** Our multi-omics approach identified a malignant epithelial subset, C5, and a five-gene signature that stratifies gastric cancer prognosis and immune response. Functional assays showed that SRI knockdown impairs tumor cell growth, migration and invasion.

## 1. Introduction

Gastric cancer ranks among the most prevalent malignancies worldwide, with an estimated one million new diagnoses and 660,000 deaths in 2022, placing it fifth in both incidence and mortality [1]. Despite advances in diagnostic and therapeutic strategies, patient outcomes remain poor, and improving survival rates continues to be a major challenge [2].

Gastric cancer’s invasive behavior and poor prognosis arise from its complex molecular biology. Tumor heterogeneity is considered a key factor influencing treatment efficacy and prognosis [3,4]. Traditional histological classifications and TNM staging often fail to capture this heterogeneity adequately, limiting their prognostic accuracy and utility for guiding personalized therapy [5,6]. Consequently, recent research has focused on uncovering molecular drivers and identifying therapeutic targets. Trastuzumab is the first validated molecular targeted drug for gastric cancer but is only effective for approximately 20% of patients with human epidermal growth factor receptor 2 (HER2) overexpression [7]. In recent years, several novel targeted agents and immunotherapeutic strategies have been introduced for gastric cancer. Notably, Monoclonal antibodies targeting programmed cell death protein 1 (PD-1) have delivered substantial clinical benefit in tumors exhibiting high levels of microsatellite instability (MSI-H) [8]. Nonetheless, gastric cancer patients lacking predictive biomarkers still exhibit a low overall response rate to immunotherapy [9]. Thus, it is imperative to identify novel actionable targets to enhance clinical outcomes for these patients.

Advancements in multi-omics approaches have shed light on novel therapeutic targets in gastric cancer. The emergence of high-resolution omics technologies, such as single-cell sequencing (scRNA-seq), has enabled researchers to analyze the tumor heterogeneity with unprecedented precision [10]. In recent years, several authoritative studies have used scRNA-seq to construct single-cell atlases of gastric cancer, revealing the transcriptional characteristics of different cell subpopulations [11,12]. Additionally, spatial transcriptomics preserves native tissue context, enabling mapping of cell–cell interactions and spatial organization that scRNA-seq alone cannot resolve [13,14].

In this study, we integrated multiple omics datasets to identify novel therapeutic targets and biomarkers in gastric cancer. Single-cell, spatial, and bulk transcriptomic profiles from multiple large-scale cohorts were leveraged to establish a comprehensive analytical framework. Using these datasets, we developed a prognostic gene signature to evaluate patient survival and compare the immune features of high- and low-risk groups. In vitro assays then confirmed the involvement of a key signature gene in promoting gastric cancer progression. These results enrich our comprehension of gastric cancer pathogenesis and could inform the design of more efficacious therapeutic strategies.

## 2. Materials and Methods

### 2.1. Data Collection

**Figure 1:**
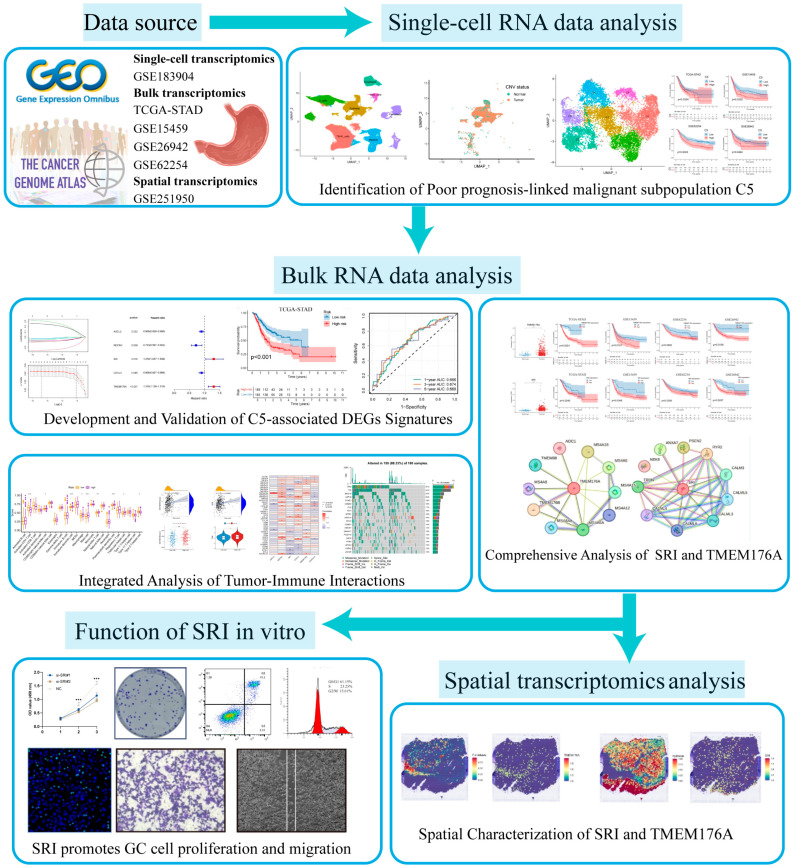
The flowchart of this study.

Data for this study were obtained from the Gene Expression Omnibus (GEO) (https://www.ncbi.nlm.nih.gov/geo/) (accessed on 25 May 2024) and The Cancer Genome Atlas (TCGA) (https://portal.gdc.cancer.gov/) (accessed on 20 May 2024). Single-cell transcriptomes were retrieved from GEO series GSE183904, comprising 26 primary gastric cancer samples. Bulk RNA-seq profiles and matched clinical metadata were retrieved from the TCGA-STAD dataset and validated across three separate GEO series (GSE15459, GSE26942, and GSE62254/ACRG). Samples with survival < 1 month were excluded from all prognostic analyses. Spatial transcriptomic data from GEO series GSE251950 were used to map in situ gene expression patterns. The overall analytical workflow is shown in Figure 1.

### 2.2. Single-Cell Data Processing

We used “seurat” package [15] to construct the single-cell object, setting the criteria that genes detected in ≥5 cells and cells expressing ≥ 300 genes. Quality control metrics included the proportions of mitochondrial (MT-) and hemoglobin (HBA/HBB) transcripts; cells with 200–6000 detected genes, 200–20,000 total counts, <20% mitochondrial reads and <5% hemoglobin reads were kept. Filtered counts were normalized using Seurat’s built-in LogNormalize method, and the variance-stabilizing transformation (VST) was used to select the top 2000 most variable genes. After standardization using ScaleData, principal component analysis (PCA) was carried out, and “Harmony” package [16] was applied to mitigate batch effects across samples. The first 30 principal components were selected to construct a nearest-neighbor graph and shared-nearest-neighbor clustering at resolution 0.8. Uniform Manifold Approximation and Projection (UMAP) was employed for visualization and cluster identities were determined from canonical cell-type marker genes.

### 2.3. Identification of Malignant Epithelial Cells Associated with Poor Prognosis

Copy number variation (CNV) inference was performed with the “infercnv” package [17]. Epithelial cells were isolated and compared against T, B, mast and endothelial cells as reference populations. Gene chromosomal coordinates (chromosomes 1–22) were retrieved via biomaRt to build the inferCNV object, and per-cell CNV scores were computed. The CNV scores of epithelial cells were then subjected to k-means clustering. Violin plots contrasted each cluster’s CNV score distribution against that of the reference group: clusters whose scores aligned with the reference were annotated as normal epithelium, while those with increased CNV scores were designated as tumor cells.

Epithelial cells identified as tumor cells were isolated into a new Seurat object. These cells were processed using the same single-cell analysis workflow as in the initial analysis. Clusters with low cell counts were removed, the shared nearest-neighbor graph was reconstructed, and UMAP was recalculated to yield malignant epithelial subpopulations. Gene Set Variation Analysis (GSVA) was applied to the Hallmark gene set (retrieved from MSigDB via msigdbr) using a Gaussian kernel to compute single-cell pathway activity scores within malignant epithelial clusters, and mean cluster-level GSVA scores were compared and displayed in a heatmap.

Non-normalized count matrices for 200 cells from each malignant epithelial subcluster were submitted to the CIBERSORTx platform (https://cibersortx.stanford.edu/) (accessed on 15 July 2024) for deconvolution. Subcluster proportions were quantified in four distinct cohorts (TCGA-STAD, GSE15459, GSE26942, and GSE62254). Finally, the association between subcluster proportions and patient survival was evaluated within each cohort.

### 2.4. Pseudotemporal Analysis and Differentiation Potential Inference

We performed pseudotime analysis on the malignant cell subpopulations using the “monocle” package [18]. Raw counts and cell metadata were combined into a CellDataSet under a negative binomial model. Size factors and dispersions were estimated, and marker genes with log_2_FC ≥ 0.25 and expression in ≥25% of cells per cluster were identified via FindAllMarkers. The top 200 genes by average log_2_FC were designated as ordering genes. Dimensionality reduction using the DDRTree algorithm produced a two-dimensional manifold along which cells were ordered in pseudotime. The resulting trajectory captures the continuous progression of malignant epithelial cells.

“CytoTRACE2” package [19] was used to estimate differentiation potential across malignant epithelial subclusters. Raw counts from the Seurat object were input to generate CytoTRACE raw scores, potency indices and relative differentiation rankings for each cell.

### 2.5. CellChat

A CellChat object was constructed from the expression profiles and metadata of malignant epithelial, immune and stromal cells, with CellChatDB.human as the ligand–receptor reference [20]. Following data filtration, upregulated genes and ligand–receptor interaction pairs were identified, and the probabilities of intercellular communication were estimated. Interactions supported by fewer than ten cells were excluded to reduce noise. Pathway-level networks were assembled from ligand–receptor probabilities, and the top four pathways exhibiting the strongest signaling intensity were chosen for subsequent network integration and functional role analysis.

### 2.6. Development and Validation of a Prognostic Signature and Clinical Nomogram

TCGA-STAD transcripts per million (TPM) data were filtered to remove genes with mean expression ≤ 1. Differential expression between GSVA and adjacent normal tissue was assessed by Wilcoxon test with FDR correction, and genes with |log_2_FC| > 1 and FDR < 0.05 were deemed significant. Upregulated tumor genes were then intersected with C5 marker genes to define C5-associated differentially expressed genes (DEGs). GO (BP, CC, MF) and KEGG enrichment analyses were performed using “ClusterProfiler” package [21] via enrichGO and enrichKEGG, with q < 0.05 indicating significant terms.

A univariate Cox proportional hazards analysis was conducted on the intersected gene set to identify prognostic candidates (*p* < 0.05). All prognostic candidates were then entered into a LASSO model with ten-fold cross-validation. Genes with nonzero coefficients at λmin were subjected to bidirectional stepwise selection to build a multivariate Cox model, yielding final regression coefficients (β), hazard ratios and *p*-values. A composite risk score was calculated as: $RiskScore=\sum_{i=1}^{n}\beta_{i}*{expr}_{i}$. Samples were dichotomized at the median score into high- and low-risk groups. The same pipeline was applied to three external cohorts (GSE84437, GSE29272 and GSE15459) for validation. Principal component analysis based on risk strata confirmed the model’s discriminative ability.

Prognostic performance was assessed by Kaplan–Meier survival curves and log-rank tests in the training set and three validation cohorts. Time-dependent ROC analysis (1-, 3- and 5-year) was conducted using the “timeROC” package [22] to calculate AUCs and quantify model discrimination over time.

A multivariate Cox model incorporating risk score, age, gender, grade and stage was used to build a nomogram via the “regplot” package. Points proportional to each coefficient were summed to estimate 1-, 3- and 5-year survival probabilities. Discrimination was assessed by time-dependent ROC curves (1-, 3- and 5-year AUCs) and calibration was performed using 1000 bootstrap iterations.

### 2.7. Immune Landscape, Checkpoint Expression, and Immunotherapy Response

Enrichment scores for immune cell and function gene sets were computed by ssGSEA from the “GSVA” package [23]. Scores were compared using Wilcoxon tests to assess associations between risk stratification and immune infiltration or functional status.

Immune checkpoint gene expression in TCGA-STAD was compared between groups using Wilcoxon tests, and genes with *p* < 0.05 were visualized by boxplots. Spearman correlations between the risk score and each checkpoint gene, as well as between checkpoint genes and signature genes, were calculated to elucidate their associations.

The ESTIMATE algorithm was applied to assess the tumor microenvironment (TME) by deriving StromalScore, ImmuneScore and the combined ESTIMATEScore for each sample. Differences in these scores were assessed by Wilcoxon tests and shown as boxplots. Spearman correlation analyses between each ESTIMATE score and the continuous risk score were calculated.

Tumor Immune Dysfunction and Exclusion (TIDE) scores for TCGA-STAD samples were obtained from the online platform (http://tide.dfci.harvard.edu/) (accessed on 3 August 2024). Group comparisons were performed using Wilcoxon tests and visualized as boxplots. The association between continuous risk score and TIDE was assessed by Spearman correlation and shown with a scatter plot.

Immunophenoscore (IPS) sub-scores for four CTLA4/PD-1 status combinations were downloaded from The Cancer Immunome Atlas (https://tcia.at/home) (accessed on 9 August 2024). Wilcoxon tests compared each sub-score between groups.

### 2.8. Drug Sensitivity Prediction and Mutational Landscape

Drug sensitivity was inferred using the “oncoPredict” framework and GDSC2 drug response data [24], Expression data were used to predict IC_50_ values for commonly used gastric cancer therapies, including 5-fluorouracil, cisplatin, paclitaxel, irinotecan and other agents. Wilcoxon tests compared predicted IC_50_ distributions between groups.

MAF files for TCGA-STAD were obtained from TCGA and stratified by risk group. We ranked genes within each group by nonsynonymous mutation frequency and visualized the top 20 per group using waterfall plots. Tumor mutational burden (TMB) distributions between groups were compared using Wilcoxon tests. Spearman correlation between continuous risk scores and TMB was calculated and visualized with a scatter plot.

### 2.9. Signature Gene Validation and Protein–Protein Interaction Analysis

Expression levels of each signature gene in adjacent normal tissues versus tumor tissues were compared in TCGA-STAD using the Wilcoxon test. Prognostic value was evaluated by Kaplan–Meier survival analysis with log-rank testing. A protein–protein interaction (PPI) network was generated from STRING (https://cn.string-db.org/) with a combined score > 0.4 (medium confidence) to explore gene interrelationships. Finally, immunohistochemical data from the Human Protein Atlas (https://www.proteinatlas.org/) (accessed on 8 October 2024) were consulted to validate differential protein expression in adjacent normal tissues and malignant gastric tissues.

### 2.10. Spatial Transcriptomics Analysis

GEO series GSE251950 was retrieved and sample GSM7990473 imported into Seurat as a Spatial assay with accompanying tissue images. SCTransform normalization was followed by PCA, adjacency graph construction, louvain clustering and UMAP embedding. Cluster assignments were projected onto tissue coordinates. Cell type labels from the single-cell Seurat object were mapped onto spatial data using FindTransferAnchors and TransferData under the same normalization framework. SpatialFeaturePlot illustrated the tissue-wide distribution of each cell type, and FeaturePlot displayed the localization patterns of key genes.

### 2.11. Cell Culture and Transfection

Human gastric cancer cell lines MGC-803 and AGS (iCell, Shanghai, China) were maintained in RPMI-1640 medium (Gibco, Thermo Fisher Scientific, Grand Island, NY, USA) supplemented with 10% fetal bovine serum (Gibco, USA) and 1% penicillin–streptomycin (Beyotime, Shanghai, China) at 37 °C in a humidified atmosphere containing 5% CO_2_. Cells were passaged at a 1:3 split ratio and transfected at 60–70% confluence using Lipofectamine™ 3000 reagent (Invitrogen, Thermo Fisher Scientific, Carlsbad, CA, USA). Complexes of siRNA (GenePharma, Shanghai, China) and Lipofectamine in Opti-MEM™ (Gibco, USA) were applied for 4–6 h, then switched to complete RPMI-1640. Cells were harvested 24–48 h post-transfection for RT-qPCR and Western blot analyses to confirm knockdown efficiency. SiRNA sequences are listed in Table S1.

### 2.12. RNA Extraction and qRT-PCR

Total RNA was extracted with FreeZol Reagent (Vazyme, Nanjing, China). 1 µg of RNA was reverse-transcribed using the HiScript III First Strand cDNA Synthesis Kit (Vazyme, China). Quantitative PCR was carried out using Taq Pro Universal SYBR qPCR Master Mix (Vazyme, China). GAPDH served as the internal reference, and relative gene expression was calculated by the 2^−ΔΔCt^ method. Primer sequences (Sangon, Shanghai, China) are provided in Table S1.

### 2.13. Western Blot

Cell lysates were prepared on ice using RIPA buffer with protease inhibitors (Beyotime, China) and clarified by centrifugation. Protein concentration was measured by BCA assay (Beyotime, China) and samples were normalized in loading buffer. Proteins were denatured at 95 °C for 10 min, resolved on 10% SDS–PAGE gels (EpiZyme, Shanghai, China) at 80–120 V, and transferred to PVDF membranes (Merck Millipore, Burlington, MA, USA) at 300 mA for 1.5–2 h. Membranes were blocked in 5% BSA/TBST (Servicebio, Wuhan, China) for 1 h at room temperature, then incubated overnight at 4 °C with anti-β-actin (Proteintech, Wuhan, China) and anti-SRI (Abcam, Waltham, MA, USA) primary antibodies in TBST. Membranes were incubated for 1 h at room temperature with rabbit IgG secondary antibody (Proteintech, China), washed again, and developed with ECL substrate (Beyotime, China) for chemiluminescence detection.

### 2.14. Cell Proliferation Assay, Colony Formation and EdU Assay

Cells plated at 5 × 10^3^ cells per well in 96-well plates. After overnight attachment, on days 1, 2 and 3 post-seeding 10 µL of CCK-8 reagent (Biosharp, Hefei, China) was added to each well. Plates were incubated at 37 °C for 1 h, and absorbance at 450 nm was recorded using a microplate reader.

Cells were seeded at 500 cells/well in 6-well plates with 2 mL complete medium and incubated at 37 °C/5% CO_2_ for 7–10 days, changing medium every 3 days. Afterward, wells were fixed in methanol for 15 min, stained with 0.5% crystal violet (Beyotime, China) for 15 min, rinsed until clear, dried, and colonies of ≥ 50 cells were counted.

Cells were seeded into 6-well plates and cultured until reaching 60–70% confluence. 10 µM EdU (APE×BIO, Houston, TX, USA) was added and incubated at 37 °C for 2 h. After PBS washes, cells were fixed in 4% paraformaldehyde for 15 min, blocked in PBS + 3% BSA, and permeabilized with 0.3% Triton X-100 (Beyotime, China) for 10 min. A Click reaction was performed per kit instructions; nuclei were counterstained with Hoechst.

### 2.15. Flow Cytometric Analysis of Cell Cycle and Apoptosis

Cell cycle analysis was performed using a PI staining kit (Beyotime, China). Cells were collected, fixed overnight at 4 °C in 70% ethanol, and subsequently incubated with RNase A and propidium iodide for 1 h in the dark. DNA content was analyzed by flow cytometry and modeled with ModFit LT.

Apoptotic rates were determined using the Annexin V-FITC/PI dual-staining kit (Beyotime, China). After PBS rinses, cells were stained with Annexin V-FITC and PI for 15 min at room temperature in the dark, then immediately analyzed by flow cytometry. Early apoptotic cells were Annexin V^+^/PI^−^ and late apoptotic cells Annexin V^+^/PI^+^. Data were quantified using FlowJo v10.9.

### 2.16. Transwell Migration and Invasion Assays

For migration, 5 × 10^4^ cells were seeded in the upper chamber; the lower chamber contained 600 μL RPMI-1640 with 20% FBS. After 24 h incubation, migrated cells were fixed in 4% paraformaldehyde for 15 min and stained with 0.1% crystal violet for 20 min. For invasion assays, Transwell inserts were coated with Matrix-Gel™ Matrigel (Beyotime, China) diluted 1:6 and incubated at 37 °C for 2 h to allow gel formation. Cells were then seeded and processed as in the migration assay.

### 2.17. Wound-Healing Assay

Log-phase cells were seeded in 6-well plates and grown to >90% confluence. A linear scratch was made with a sterile 200 µL pipette tip, wells were washed twice with PBS to remove debris, and serum-reduced medium containing 1% FBS was added. Images of the wound area were captured immediately (0 h) and at 24 h and 48 h using an inverted microscope. Wound widths were measured in ImageJ 1.53q.

## 3. Results

### 3.1. Single-Cell Transcriptomic Atlas of Gastric Cancer

**Figure 2:**
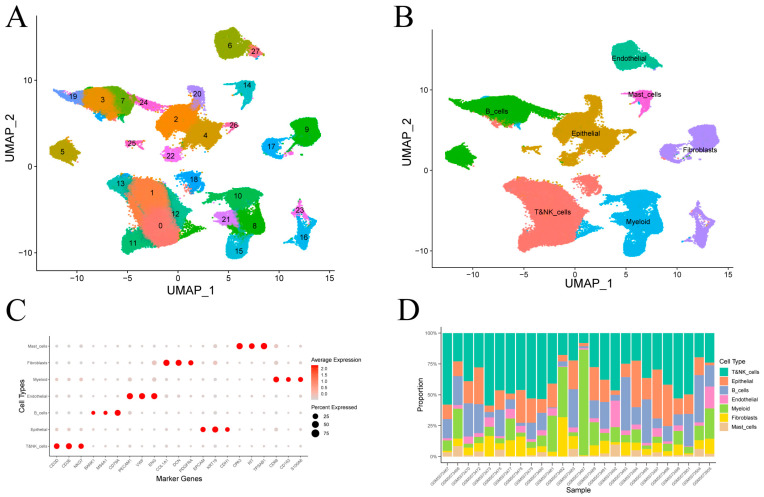
Clustering and annotation of gastric cancer single-cell transcriptomes. (**A**) Visualization of 28 transcriptional clusters. (**B**) Assignment of clusters to seven cell types using canonical marker genes. (**C**) Average expression and fraction of positive cells for representative markers in each annotated cell type. (**D**) Cell-type proportions across individual samples.

After quality control of single-cell transcriptomes from 26 gastric cancer samples (Figure S1), clustering revealed 28 transcriptional subpopulations (Figure 2A). These were assigned to seven major cell types—epithelial cells, T and NK cells, B cells, fibroblasts, endothelial cells, myeloid cells and mast cells—based on canonical marker expression (Figure 2B). Dot plot visualizations confirmed annotation accuracy (Figure 2C). Epithelial and immune lineages (T/NK, B, and myeloid cells) comprised the majority of cells in most tumor samples (Figure 2D).

### 3.2. Inference of Malignant Epithelial Cells and Identification of the C5 Subcluster Associated with Poor Prognosis

**Figure 3:**
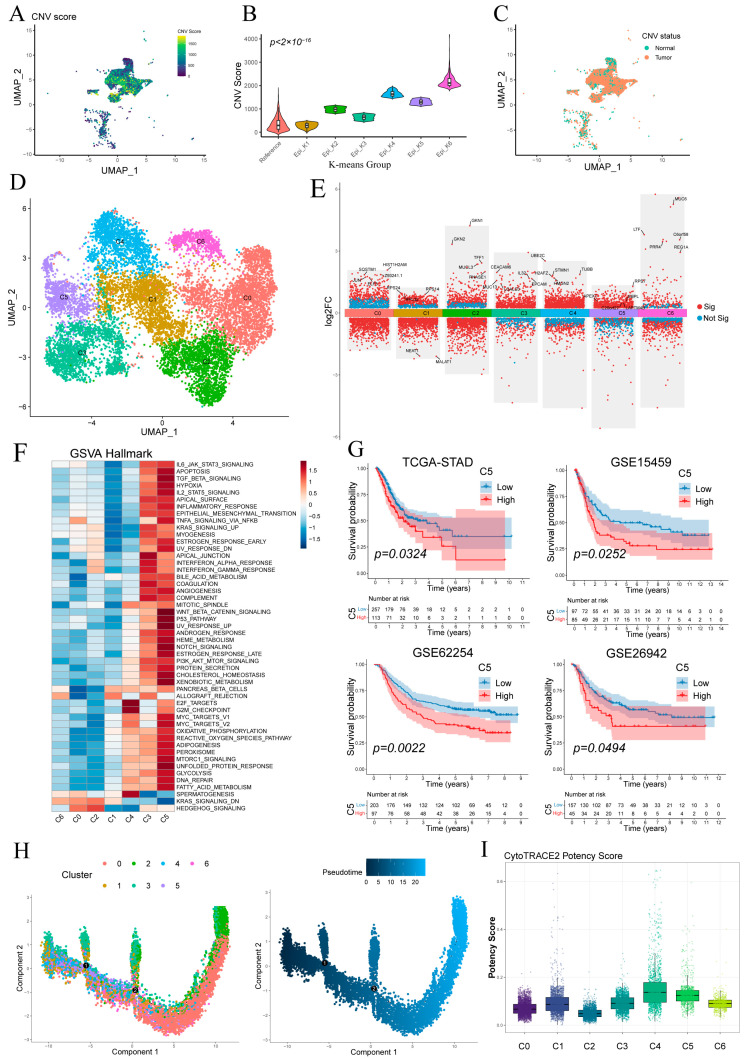
Stratification and functional characterization of epithelial subpopulations. (**A**) Epithelial cells colored by single-cell copy number variation (CNV) score. (**B**) CNV score distributions of epithelial clusters defined by k-means. (**C**) Normal and malignant epithelial subsets defined by per-cell CNV scores. (**D**) Malignant epithelial cells reclustered into six malignant subpopulations (C0–C5). (**E**) Volcano plot of differentially expressed genes in malignant epithelial cells. (**F**) Heatmap of pathway activity scores across malignant subclusters. (**G**) Kaplan–Meier survival curves showing that high abundance of subcluster C5 correlates with worse overall survival in four cohorts. (**H**) Monocle pseudotime trajectory of malignant epithelial cells, colored by subcluster identity (left) and inferred differentiation pseudotime (middle). (**I**) Boxplot of CytoTRACE potency scores for each malignant subcluster.

Figure 3A shows the UMAP visualization of single-cell CNV scores for epithelial cells, and most epithelial cells exhibiting more pronounced CNV compared to the reference group (Figure S2). K-means clustering separated epithelial cells into six groups, with epi_k1—whose CNV profile matched the reference—classified as “Normal” and the other five clusters as “Tumor” (Figure 3B,C). Tumor cells were then re-clustered into six malignant subpopulations (C0–C5) (Figure 3D), and their key differentially expressed genes are detailed in Figure 3E.

GSVA revealed distinct pathway activations across malignant subclusters (Figure 3F). C5 showed broad enrichment of oncogenic/inflammatory signaling (WNT/β-catenin, p53, Notch, IL-2/STAT5, TNFα/NF-κB) and metabolic activation (glycolysis, fatty acid metabolism, OXPHOS), indicative of inflammatory and invasive potential. C3 was preferentially associated with KRAS signaling, apical junction formation and interferon-α/γ responses. C4 exhibited predominant enrichment of cell cycle-associated pathways, notably E2F target genes and the G2/M checkpoint. Other clusters lacked significant hallmark enrichment.

Kaplan–Meier analysis demonstrated that high C5 abundance predicted significantly poorer overall survival (Figure 3G), whereas C3 levels showed no significant prognostic association (Figure S3A).

Monocle2 pseudotime ordering (Figure 3H) revealed a linear progression from C4/C5/C3 toward C0/C1, indicating a continuum of malignant evolution. CytoTRACE analysis showed C5 and C4 exhibiting relatively higher stemness scores and C0/C1 the lowest, consistent with pseudotime findings and supporting C5 as a less differentiated, highly proliferative subset (Figure 3G and Figure S3B).

### 3.3. Cell–Cell Communication Between Epithelial Subclusters and the Tumor Microenvironment

**Figure 4:**
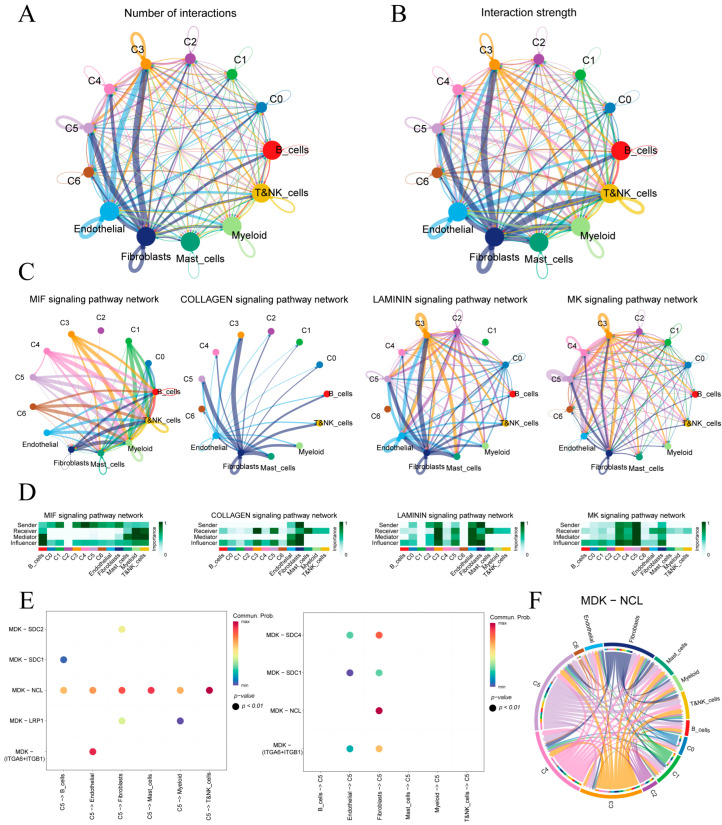
Cell–cell communication in the gastric tumor microenvironment. (**A**,**B**) Circular plots of interaction number and strength among epithelial subclusters and immune/stromal cells. (**C**) Top four signaling pathways ranked by overall communication strength. (**D**) Heatmaps of pathway-specific roles for each cell type in the four highlighted pathways. (**E**) Bubble plot of MK pathway sending and receiving by subcluster C5. (**F**) Circos diagram of MDK–NCL ligand–receptor links across all cell populations.

Figure 4A,B quantify ligand–receptor interaction counts and strengths between malignant epithelial subclusters (C0–C5) and immune or stromal cells. Subclusters C3 and C5 exhibit the highest number and intensity of interactions, underscoring their prominent roles in tumor–microenvironment crosstalk.

Four key signaling pathways—MIF, COLLAGEN, LAMININ and MK—were identified based on overall communication strength (Figure 4C). In the MIF network, malignant epithelial cells predominantly signal to immune cells, with myeloid and T/NK cells as principal receptors. COLLAGEN signaling is stromal-centric, driven mainly by fibroblasts toward epithelial subclusters. LAMININ interactions center on endothelial–fibroblast crosstalk and include notable autocrine loops in C3. MK shows extensive epithelial–stromal interactions. A heatmap of sender, mediator and receiver scores (Figure 4D) confirms that fibroblasts lead ECM-related communications (COLLAGEN, LAMININ). C4 showed the highest MIF output targeting lymphocytes, whereas C5 dominated MK signaling with the greatest sending and influence scores.

We further analyzed the cell communication between C5 and its tumor microenvironment (Figure S4). Given C5’s pivotal involvement in the MK signaling cascade, we examined its principal ligand–receptor interactions (Figure 4E). In the MK pathway, C5 primarily signals through the MDK–NCL ligand–receptor axis and, when acting as a receiver, most MDK ligands originate from epithelial cells and fibroblasts. Figure 4F maps the MDK–NCL network, showing fibroblasts and endothelial cells as hubs and epithelial subclusters C5, C4 and C3 as key senders and receivers, underscoring the MK axis’s central role in epithelial–stromal–immune crosstalk.

### 3.4. Identification and Enrichment Analysis of C5-Associated Upregulated DEGs

**Figure 5:**
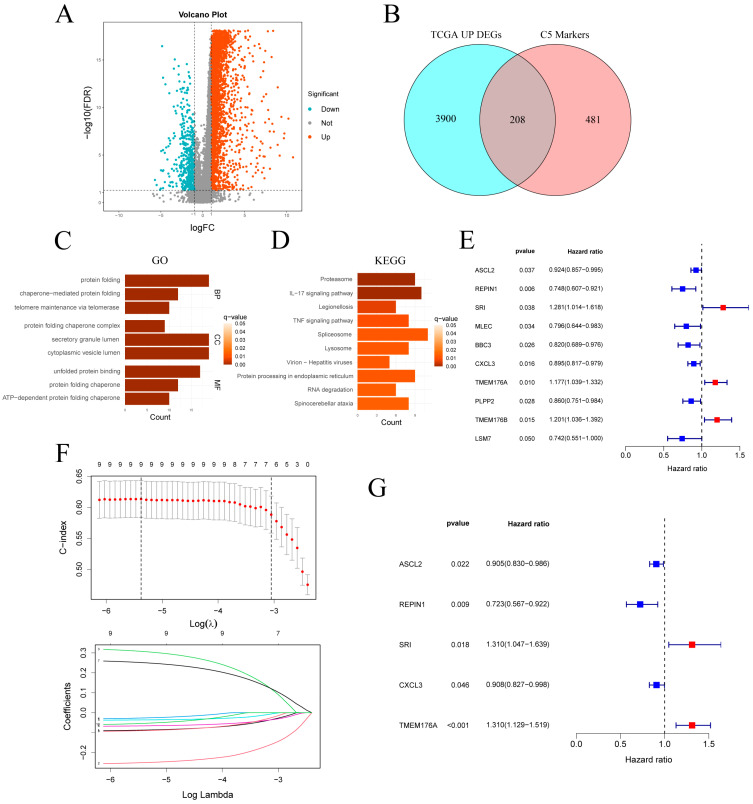
Identification and validation of a C5-derived prognostic gene signature. (**A**) Volcano plot of DEGs in TCGA-STAD (|log2FC| > 1, FDR < 0.05). (**B**) Venn diagram intersecting TCGA up-regulated DEGs with C5 marker genes. (**C**,**D**) GO and KEGG enrichment analyses of the intersected gene set. (**E**) Univariate Cox regression of the intersected genes. (**F**) LASSO regression selecting key prognostic genes. (**G**) Forest plot of multivariate Cox analysis for the selected signature genes. TCGA-STAD: The Cancer Genome Atlas Stomach Adenocarcinoma; DEGs: differentially expressed genes.

In the TCGA-STAD cohort, differential expression analysis revealed 4108 upregulated and 483 downregulated genes (Figure 5A). Intersection of the 4108 upregulated genes with the 689 C5 marker genes yielded 208 overlapping genes (Figure 5B). GO enrichment of the genes (Figure 5C) highlighted pathways related to protein homeostasis and quality control, including protein folding, chaperone-mediated folding, telomere maintenance and secretory granule lumen. KEGG analysis (Figure 5D) revealed significant enrichment in the proteasome, IL-17 and TNF signaling pathways, and protein processing in the endoplasmic reticulum. Additional enrichment in spliceosome and RNA degradation pathways suggests elevated RNA turnover in C5 cells. Together, these findings indicate that C5 subcluster cells are characterized by enhanced protein metabolism, inflammatory signaling and RNA processing.

### 3.5. Construction and Validation of a C5-Associated Gene Signature and Clinical Nomogram

Univariate Cox analysis of the intersected genes (Figure 5E) identified 10 survival-related candidates: ASCL2, REPIN1, MLEC, BBC3, CXCL3, PLPP2 and LSM7 were protective, whereas SRI, TMEM176A and TMEM176B conferred increased risk. LASSO regression retained nine genes at the optimal λ (Figure 5F), and bidirectional stepwise selection further narrowed this to five for multivariate Cox modeling (Figure 5G). In the final model, ASCL2 (HR = 0.905, *p* = 0.022), REPIN1 (HR = 0.723, *p* = 0.009) and CXCL3 (HR = 0.908, *p* = 0.046) remained protective, while SRI (HR = 1.310, *p* = 0.018) and TMEM176A (HR = 1.310, *p* < 0.001) emerged as independent risk factors.

**Figure 6:**
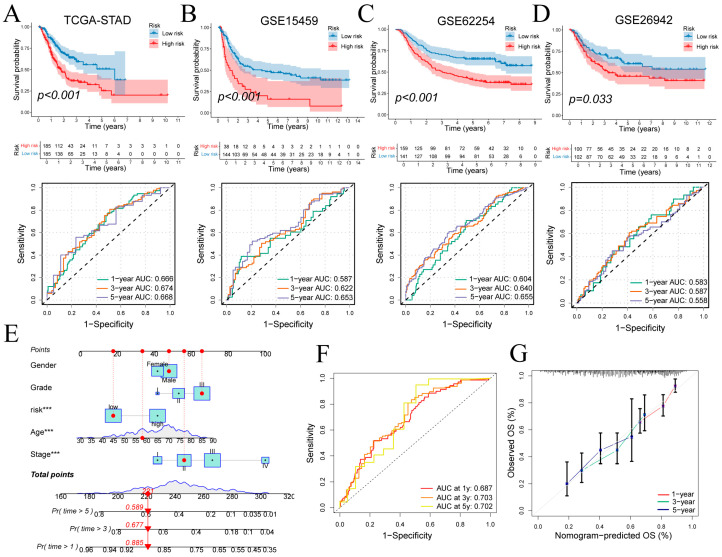
Prognostic performance of the gene signature and nomogram. (**A**–**D**) Kaplan–Meier curves for high- versus low-risk groups and time-dependent ROC curves in four cohorts. (**E**) Nomogram integrating risk score and clinical covariates for survival prediction. (**F**,**G**) ROC and calibration curves evaluating nomogram performance at 1, 3 and 5 years. ROC: Receiver Operating Characteristic. Wilcoxon test *** *p* < 0.001.

Patients were stratified into high- and low-risk groups based on signature-derived risk scores in TCGA-STAD. Kaplan–Meier analysis demonstrated significantly poorer overall survival in the high-risk cohort (log-rank *p* < 0.001; Figure 6A top), and time-dependent ROC curves yielded AUCs of 0.666, 0.674 and 0.668 at 1, 3 and 5 years (Figure 6A bottom). Risk status correlated with T, N and overall stage (Figure S3C). In external cohorts, high-risk status again predicted adverse outcomes: GSE15459 (*p* < 0.001; 1/3/5-year AUCs = 0.587, 0.622, 0.653; Figure 6B), GSE62254 (*p* < 0.001; AUCs = 0.604, 0.640, 0.655; Figure 6C) and GSE26942 (*p* = 0.033; AUCs = 0.583, 0.587, 0.558; Figure 6D). These findings confirm the signature’s reasonable prognostic performance and generalizability.

A multivariate Cox regression incorporating risk score, age, sex, tumor grade, and clinical stage was used to construct the nomogram (Figure 6E). Discriminative ability was evaluated by time-dependent ROC analysis of total nomogram points, yielding 1-, 3- and 5-year AUCs of 0.687, 0.703 and 0.702, respectively (Figure 6F). Calibration curves closely matched the ideal diagonal at all three time points, confirming strong agreement between predicted and observed survival (Figure 6G).

### 3.6. Immune Infiltration, Checkpoint Profiles and Tumor Microenvironment Features

**Figure 7:**
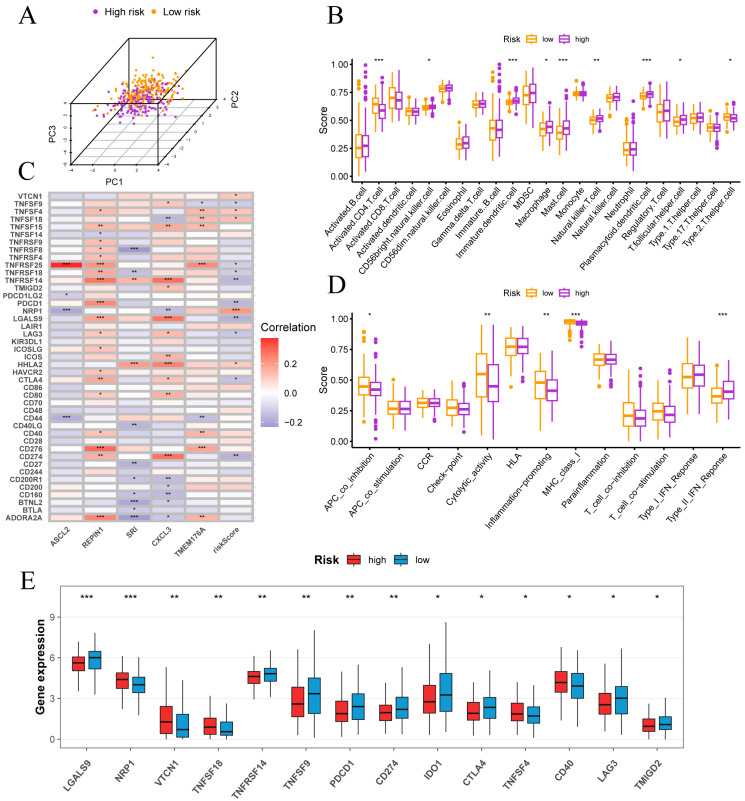
Immune infiltration and checkpoint features in high- and low-risk groups. (**A**) Separation of high- and low-risk samples in principal component space. (**B**) Correlations between risk score and immune cell infiltration levels. (**C**) Correlations between risk score, signature genes and key immune checkpoint genes. (**D**) Associations of risk scores with immune functional pathways. (**E**) Comparison of immune checkpoint gene expression between risk groups. Normality assessed by Shapiro–Wilk test. Wilcoxon test * *p* < 0.05, ** *p* < 0.01, *** *p* < 0.001.

In PCA space, high- and low-risk samples exhibited distinct clustering (Figure 7A). ssGSEA of 28 immune cell types revealed that high-risk tumors were enriched for CD56bright NK cells, immature dendritic cells, macrophages, mast cells, NKT cells, plasmacytoid dendritic cells and T follicular helper cells, whereas low-risk tumors showed higher activated CD4 T cell and Th2 cell infiltration (Figure 7B). Functional ssGSEA indicated that low-risk samples had elevated APC co-inhibition, cytolytic activity, inflammation-promoting and MHC-I scores, while high-risk tumors displayed a pronounced type II interferon response (Figure 7D).

Figure 7C reveals that risk score correlates inversely with PD-L1 (CD274), PD-1 (PDCD1), CTLA4, LAG3, LGALS9, TNFRSF14, TNFRSF18, TNFRSF25 and TNFSF9, but positively with VTCN1 (B7-H4), TNFSF4 (OX40L), TNFSF18 (GITRL), NRP1 and HHLA2. Among signature genes, REPIN1 and TMEM176A show positive associations with most checkpoints, SRI is largely negative and ASCL2 is notably positive with TNFRSF25. Consistent with these correlations, Figure 7E shows higher expression of PD-1, PD-L1, CTLA4 and LAG3 in the low-risk group, whereas the high-risk group exhibits elevated VTCN1, NRP1, TNFSF18 and CD40. These data indicate that classic checkpoint inhibitors may be more effective in low-risk patients.

**Figure 8:**
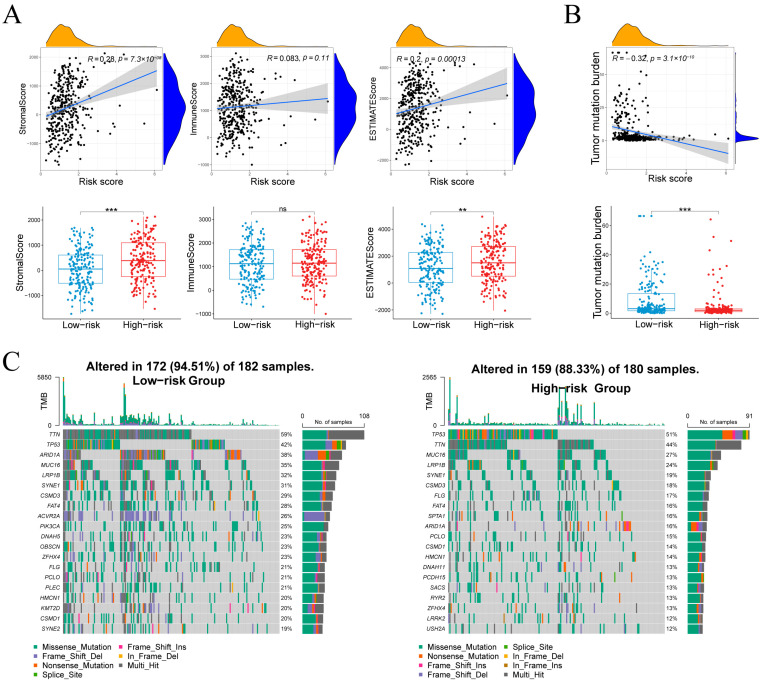
Associations between the risk score and microenvironment scores, tumor mutational burden, and the mutational landscape. (**A**) Stromal, immune and composite scores in relation to risk score and group. (**B**) Relationship between tumor mutational burden and risk score. (**C**) Waterfall plots of the 20 most frequently mutated genes in each risk cohort. Normality assessed by Shapiro–Wilk test. Wilcoxon test ** *p* < 0.01, *** *p* < 0.001; ns not significant.

Risk score correlated with StromalScore (r = 0.28, *p* = 7.3 × 10^−8^) and ESTIMATEScore (r = 0.20, *p* = 1.3 × 10^−4^), but not ImmuneScore (r = 0.08, *p* = 0.11; Figure 8A), indicating that high-risk tumors harbor more stromal content without increased immune infiltration. TMB showed an inverse relationship with risk score (r = –0.32, *p* = 3.1 × 10^−10^; Figure 8B), and was higher in the low-risk group, suggesting greater neoantigen load in these tumors.

### 3.7. Mutation Landscapes

Figure 8C compares somatic mutation landscapes between groups. In the low-risk cohort, 94.5% of samples (172/182) harbored mutations across a wide spectrum, most frequently TTN (59%), TP53 (42%), ARID1A (38%), MUC16 (35%), LRP1B (32%), SYNE1 (31%), CSMD3 (28%) and FAT4 (28%). By contrast, 88.3% of high-risk samples (159/180) were mutated, dominated by TP53 (51%) and TTN (44%), followed by MUC16 (27%), LRP1B (24%), SYNE1 (19%) and CSMD3 (18%), with additional recurrent changes in SPTA1, DNAH11, PCDH15, SACS, RYR2, LRRK2 and USH2A. Both groups exhibited predominantly missense mutations alongside splice-site and frameshift variants, reflecting the low-risk group’s higher TMB and broader mutational spectrum.

### 3.8. Immune Escape, Drug Sensitivity, and Immunophenoscore Analysis

**Figure 9:**
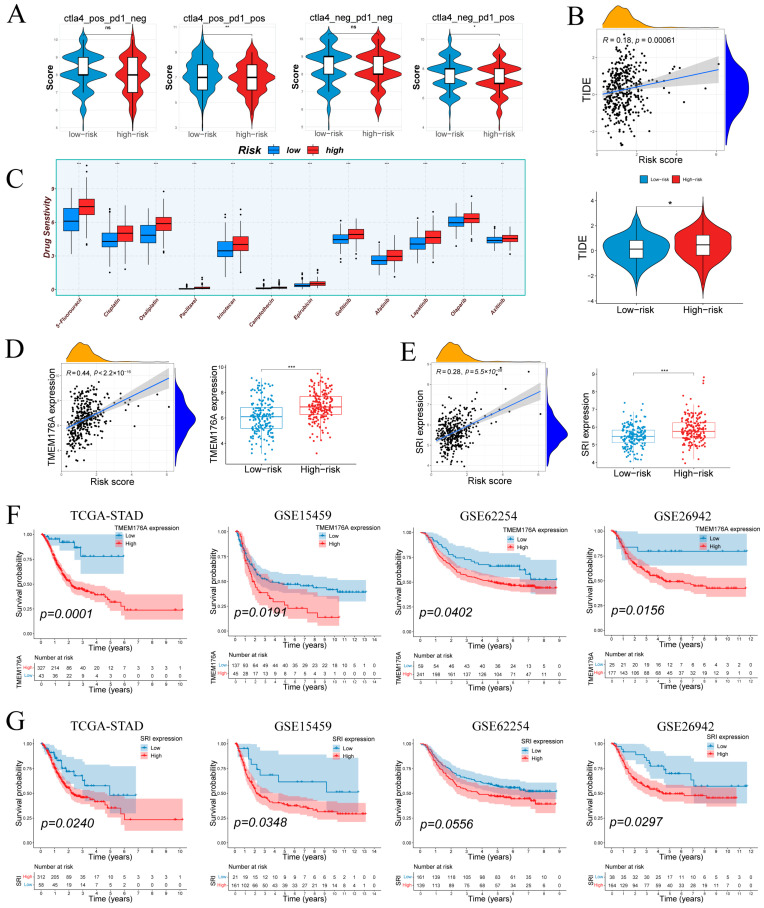
Therapeutic response by risk group; prognostic associations of TMEM176A/SRI. (**A**) Immunophenoscore differences across subtypes by risk group. (**B**) Tumor Immune Dysfunction and Exclusion (TIDE) scores in relation to risk score and group. (**C**) Differential drug sensitivity between low- and high-risk patients. (**D**,**E**) TMEM176A and SRI expression positively correlate with risk score and are elevated in the high-risk group. (**F**,**G**) TMEM176A and SRI associates with poorer survival in four cohorts. Normality assessed by Shapiro–Wilk test. Wilcoxon test * *p* < 0.05, ** *p* < 0.01, *** *p* < 0.001; ns not significant.

IPS analysis (Figure 9A) showed no difference under CTLA4^−^/PD1^−^ or CTLA4^+^/PD1^−^ conditions but revealed higher IPS in low-risk tumors with PD-1 blockade (CTLA4^−^/PD1^+^ and CTLA4^+^/PD1^+^), suggesting greater predicted benefit from anti–PD-1 therapies in low-risk group. TIDE scores correlated positively with risk (r = 0.18, *p* = 6.1 × 10^−4^; Figure 9B), and high-risk tumors exhibited significantly elevated TIDE values compared to low-risk counterparts. Drug sensitivity predictions (Figure 9C) indicated that high-risk samples were more responsive to standard chemotherapeutics (5-FU, cisplatin, oxaliplatin, paclitaxel, irinotecan, camptothecin, epirubicin) and EGFR/HER inhibitors (gefitinib, afatinib, lapatinib), as well as the VEGFR inhibitor axitinib.

Figure S5A shows that the SRI-high group was more sensitive to DNA-damage and replication-stress therapies as well as IGF-1R, HDAC, and mTOR blockade, whereas the SRI-low group was more sensitive to inhibition of MAPK/ERK, MET/VEGFR, WEE1/PIM, and the NEDD8-activating enzyme. In parallel, predicted immunotherapy response did not differ by SRI on TIDE (Figure S5B), while IPS was higher in SRI-low under PD1-positive conditions and unchanged under PD1-negative settings (Figure S5C).

### 3.9. Tumor-Specific Upregulation and Prognostic Relevance of TMEM176A and SRI

TMEM176A and SRI were prioritized for validation due to their association with poor prognosis. Both genes correlated positively with risk score and were significantly overexpressed in high-risk samples (Figure 9D,E). Kaplan–Meier analyses showed that elevated TMEM176A predicted worse overall survival in TCGA-STAD (*p* = 0.0001) and three external cohorts (GSE15459: *p* = 0.0191; GSE62254: *p* = 0.0402; GSE26942: *p* = 0.0156; Figure 9F). High SRI expression similarly conferred poor prognosis in TCGA-STAD (*p* = 0.0240), GSE15459 (*p* = 0.0348) and GSE26942 (*p* = 0.0297), with a trend in GSE62254 (*p* = 0.0556; Figure 9G). These results confirm TMEM176A and SRI as upregulated, risk-associated markers in gastric cancer.

**Figure 10:**
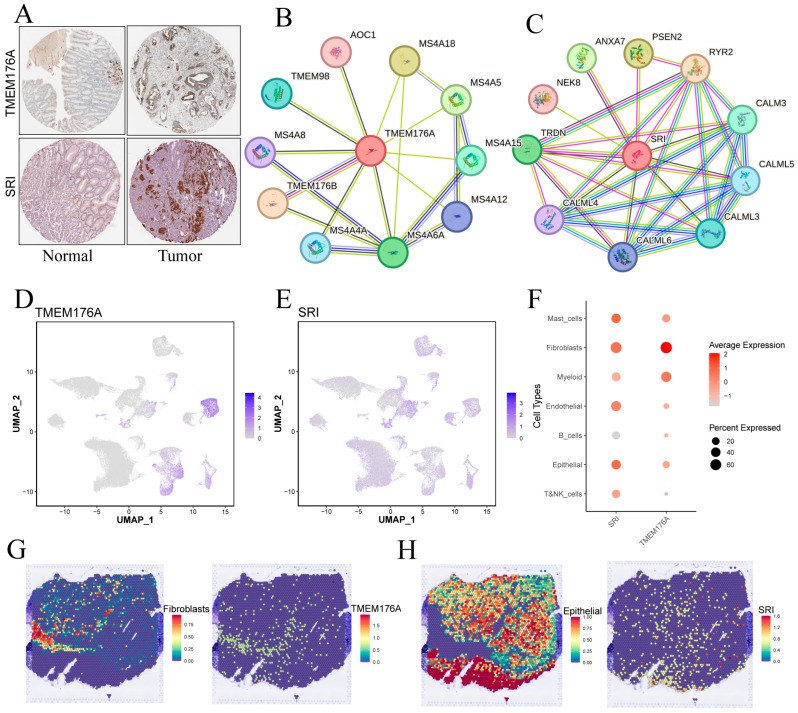
Validation and cellular localization of TMEM176A and SRI. (**A**) IHC images from the Human Protein Atlas showing TMEM176A and SRI staining in normal gastric mucosa and tumor tissue. (**B**,**C**) Protein–protein interaction networks centered on TMEM176A and SRI. (**D**,**E**) Single-cell expression levels for TMEM176A and SRI. (**F**) Dot plot of TMEM176A and SRI expression across major cell types. (**G**,**H**) Spatial transcriptomic maps displaying TMEM176A (**G**) and SRI (**H**) expression patterns within tumor sections.

HPA immunohistochemistry confirmed higher TMEM176A and SRI protein levels in gastric cancer versus adjacent mucosa (Figure 10A). TMEM176A’s PPI network is dominated by membrane-associated partners, including MS4A family members, TMEM176B, TMEM98 and AOC1, suggesting roles in membrane microdomains, receptor signaling and vesicular transport (Figure 10B). SRI’s network features calmodulins (CALM3, CALML3–6), RYR2, TRDN and membrane dynamics regulators (ANXA7, PSEN2, NEK8), pointing to functions in Ca^2+^ homeostasis and intracellular membrane system signaling (Figure 10C).

### 3.10. Single-Cell and Spatial Mapping of TMEM176A and SRI Distributions

UMAP feature plots (Figure 10D,F) reveal that TMEM176A expression is highest in fibroblasts, with lower signals in mast, myeloid and epithelial cells, whereas SRI displays moderate, widespread expression across epithelial, fibroblast and endothelial populations. Spatial transcriptomics (Figure 10G,H) corroborates these findings: TMEM176A localizes to fibroblast-rich regions, whereas SRI overlaps chiefly with epithelial areas, suggesting that TMEM176A is stroma-enriched and SRI is epithelium-enriched.

### 3.11. SRI Knockdown Impairs Proliferation and Migration In Vitro

**Figure 11:**
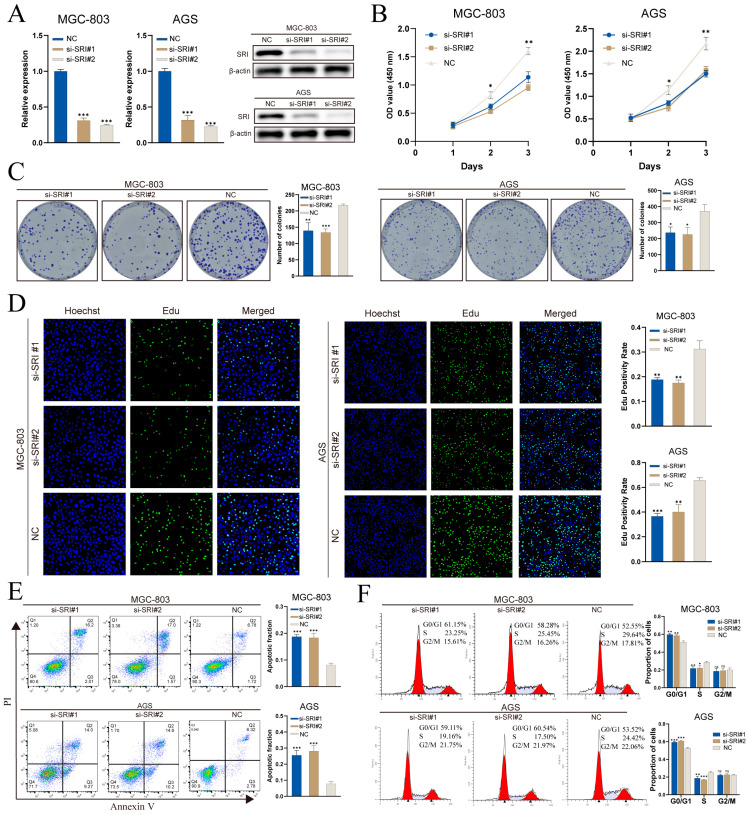
SRI knockdown impairs gastric cancer cell proliferation and induces apoptosis. (**A**) qRT-PCR and Western blot confirm siRNA-mediated SRI silencing. (**B**) CCK-8 assays show decreased viability in si-SRI cells. (**C**) Colony formation is reduced after SRI knockdown. (**D**) EdU assays reveal lower DNA synthesis in si-SRI groups. (**E**) Annexin V/PI flow cytometry indicates increased apoptosis upon SRI depletion. (**F**) PI staining demonstrates S-phase cell cycle arrest in si-SRI cells. Bars represent mean ± SEM; Student’s *t*-test * *p* < 0.05, ** *p* < 0.01, *** *p* < 0.001; ns not significant.

Spatial transcriptomics indicates that TMEM176A expression is predominantly localized to fibroblasts, whereas conventional gastric cancer cell lines are epithelial and lack a stromal component, limiting functional assessment in vitro. By contrast, SRI is upregulated in malignant epithelium and correlates with poor prognosis, making it a suitable target for cell-based validation. In MGC-803 and AGS cells, two independent siRNAs (si-SRI#1 and si-SRI#2) achieved SRI knockdown at both mRNA and protein levels, as demonstrated by RT-qPCR and Western blot (Figure 11A).

CCK-8 assays demonstrated that SRI knockdown impaired proliferation, with OD_450_ readings significantly reduced on days 2 and 3 versus controls (Figure 11B). Colony-formation assays revealed fewer and smaller crystal violet–stained colonies in both MGC-803 and AGS cells following SRI silencing (Figure 11C). EdU incorporation was substantially diminished after knockdown, indicating reduced S-phase entry and DNA synthesis (Figure 11D). Annexin V/PI flow cytometry analysis revealed a marked increase in the proportion of apoptotic cells (Q2 + Q3) after SRI knockdown (Figure 11E). Cell-cycle analysis further revealed G_0_/G_1_ arrest and a concomitant decrease in S-phase fraction, with no notable change in G_2_/M (Figure 11F).

**Figure 12:**
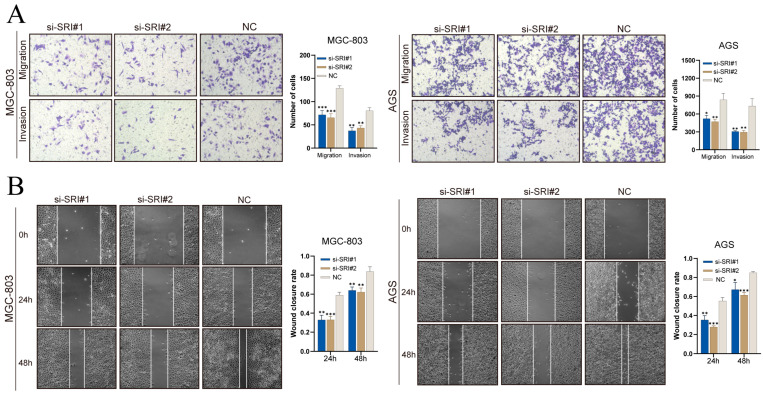
SRI knockdown reduces gastric cancer cell migration and invasion. (**A**) Transwell assays showing fewer migrated and invaded gastric cancer cells after SRI silencing. (**B**) Wound-healing assays reveal reduced closure rates at 24 and 48 h in si-SRI cells. Bars represent mean ± SEM; Student’s *t*-test * *p* < 0.05, ** *p* < 0.01, *** *p* < 0.001.

Transwell assays (Figure 12A) showed that SRI silencing decreased the numbers of MGC-803 and AGS cells migrating through pores and invading through Matrigel. In scratch assays (Figure 12B), SRI knockdown significantly reduced wound closure at 24 h and 48 h. These results demonstrate that SRI downregulation impairs gastric cancer cell migration and invasion.

## 4. Discussion

Early detection of gastric cancer remains challenging, and frequent recurrence and metastasis drive its poor prognosis [25]. The intricate molecular underpinnings of tumor initiation and progression—encompassing intratumoral heterogeneity, somatic mutation landscapes and microenvironmental interactions—are incompletely defined [26]. Despite rapid advances in single-cell and spatial transcriptomics that have sharpened our understanding of these contributing factors, each modality has inherent limitations [10]. Single-cell RNA sequencing offers cellular resolution but loses in situ spatial context, whereas spatial transcriptomics preserves tissue architecture yet often aggregates transcripts from multiple cells per capture spot and exhibits lower mRNA detection efficiency, yielding mixed signals at gene level [27,28]. Accordingly, Integrative, multi-level analyses that dissect mechanisms of gastric cancer and translate findings into targeted interventions are essential for advancing precision therapies and ultimately improving patient outcomes.

Tumor epithelial cells and their surrounding microenvironment engage in a reciprocal remodeling network. Through epithelial–mesenchymal transition (EMT) and phenotypic plasticity make cancer cells more invasive and able to evade immunity, while their cytokines and exosomes reprogram nearby stromal and immune cells [29]. In turn, stromal and immune cells remodel the extracellular matrix (ECM), alter metabolism and activate immunosuppression. These changes reinforce epithelial stemness, drug resistance, and immune exclusion, sustaining a “cold” tumor microenvironment [30,31,32]. Molecules governing these epithelial–stromal interactions represent pivotal targets for modulating immunotherapy and combination strategies as well as for refining prognostic stratification [33].

In this study, multi-omics integration pinpointed a poorly differentiated malignant epithelial subset, C5, as a central driver of gastric cancer aggressiveness. GSVA revealed C5 enrichment in oncogenic (Wnt/β-catenin, p53, Notch), inflammatory and metabolic pathways linked to adverse outcomes. Pseudotime and CytoTRACE confirmed its low differentiation status. Cell–cell interaction analysis showed C5 engaging predominantly with fibroblasts and endothelial cells via MDK–NCL axes. Then, a prognostic signature derived from C5-associated upregulated genes was validated across four independent cohorts, with low-risk patients showing more favorable survival and high-risk patients the opposite. The nomogram also performed well. Nevertheless, the signature assumes linear, proportional hazards and may miss nonlinear effects and gene–gene interactions, and the absence of treatment regimens and molecular subtypes may limit predictive accuracy. Looking ahead, the use of machine-learning models that capture nonlinear relationships, coupled with richer patient-level data, could further enhance predictive accuracy.

The high- and low-risk groups, as defined by this signature, exhibit a series of differences in immune characteristics. In the immune infiltration analysis, the high-risk group had higher infiltration scores in subpopulations such as CD56bright. NK cells, Immature dendritic cells, Macrophages, Mast cells, NK T cells, Plasmacytoid dendritic cells, and T follicular helper cells. These cells have weak direct tumor-killing effects but strong chemokine and inflammatory mediator secretion abilities [34,35,36,37,38]. Such a cellular composition may lead to an immune microenvironment primarily driven by inflammation-mediated regulation rather than effective cytotoxic responses, which could be exploited by the tumor to promote immune tolerance or immune escape [39]. The low-risk group showed higher scores in Activated CD4 T cells and Type 2 T helper cells. Activated CD4 T cells play a key role in tumor immunity by producing cytokines that activate M1 macrophages and enhance CD8^+^ T-cell cytotoxicity [40,41]. Th2 cells have dual roles in tumor immunity. They can suppress Th1 responses via IL-4 and polarize macrophages toward an M2 phenotype, promoting immune evasion [42,43]. Under other conditions, IL-5 from Th2 cells activates eosinophils and macrophages, yielding anti-tumor activity and growth inhibition [44,45]. Overall, the low-risk group is dominated by helper T cell activation. An augmented T cell-mediated antitumor immune response could partly account for the superior prognosis observed in the low-risk cohort.

Immunotherapy research in gastric cancer has focused on typical immune checkpoint pathways such as PD-1/PD-L1, CTLA-4, and LAG-3. In advanced gastric cancer, immune checkpoint inhibitors targeting PD-1/PD-L1 have become part of standard therapy [46,47]. PD-L1 expression levels are commonly used to predict the response to immunotherapy and serve as important companion diagnostic markers [48]. The CTLA-4 pathway also plays a role in regulating anti-tumor immunity, and its combination with PD-1 inhibitors has shown promising therapeutic potential [49,50]. LAG-3, an emerging immune checkpoint molecule, is often co-expressed with PD-1 in infiltrating lymphocytes of gastric cancer, and its high expression is frequently accompanied by upregulation of PD-L1 and CTLA-4, as well as higher TMB [51]. In this study, the low-risk group showed significantly higher expression in several inhibitory immune checkpoint axes, including PDCD1 (PD-1), CD274 (PD-L1), CTLA4, and LAG3. In contrast, high-risk samples did not show upregulation of the classical adaptive inhibitory axes but tended to express non-classical B7 family inhibitors like B7-H4/HHLA2 and co-stimulatory ligands like OX40L/GITRL. IPS analysis also indicated that the low-risk group had higher predicted sensitivity to PD-1-containing immunotherapy. In TIDE analysis, the high-risk group had higher scores. This pattern reflects stronger T-cell dysfunction or exclusion and implies a lower chance of responding to immune checkpoint inhibitors [52]. Notably, the correlation between the risk score and TIDE was small (R = 0.18), indicating a modest, directionally consistent association rather than strong predictive power for TIDE. These results suggest that the high-risk group may have a stronger or more unique immune evasion phenotype, leading to poor responses to classic immunotherapy.

Correlation analysis further revealed that TMEM176A is positively correlated with most immune checkpoints, potentially serving as a molecular indicator of a “checkpoint-enriched” state, while SRI is negatively correlated with most immune checkpoints, indicating a “checkpoint-low expression” phenotype.

TMEM176A, a member of the MS4A superfamily, functions as a transmembrane cation channel highly expressed in myeloid lineages [53]. Together with its paralog TMEM176B, it maintains dendritic cells in an immature, tolerogenic state and promotes MHC class II antigen presentation to naïve CD4 T cells [54,55,56]. In tumors, TMEM176A is overexpressed across diverse malignancies, including breast, liver, and skin cancers as well as lymphoid neoplasms [57,58]. TMEM176A may have different functions in various tumor types; studies have shown that indicates that it can trigger apoptotic cell death and inhibit their migration and invasion in colorectal cancer cells [59], while other research suggests that it acts as a tumor promoter in glioblastoma cells, potentially promoting progression through ERK1/2 phosphorylation [57]. Although research on TMEM176A in gastric cancer is limited, our single-cell and spatial transcriptomic analyses localize TMEM176A expression predominantly to fibroblasts and myeloid cells. Clinically, elevated TMEM176A correlates with poorer survival across several cohorts. These observations implicate TMEM176A as a stromal mediator of tumor progression in gastric cancer, and dedicated functional studies are needed to define its precise role.

Sorcin (SRI) is a soluble calcium-binding protein frequently overexpressed across diverse malignancies [60,61,62], where it drives tumor progression and mediates multidrug resistance (MDR) [63,64]. Our analyses also indicated that SRI expression stratifies sensitivity to multiple drugs, and IPS analysis suggested better PD-1–blockade responsiveness in the SRI-low group. Studies have shown that Sorcin can stimulate the EMT process in colorectal cancer via activation of the PI3K/Akt pathway [65]. It also interacts with the Smad4/ZEB1 and miR-142-5p loops, mediating paclitaxel resistance and stemness in ovarian cancer [66]. In gastric cancer, one study demonstrated that its overexpression confers MDR in gastric cancer cells [63]. Another study indicated that Sorcin promotes migration and invasion and aids in STAT3 activation in gastric cancer cells [67]. Our integrative single-cell and spatial transcriptomic analyses demonstrate that SRI is broadly expressed across epithelial, fibroblast and endothelial compartments within gastric tumors, and consistently correlates with poorer survival. Functional validation in vitro further revealed that SRI silencing provokes G_1_/S arrest and apoptosis, attenuating tumor cell proliferation as well as migratory and invasive behaviors. These findings implicate SRI as a multifaceted driver of gastric cancer progression and a promising target for therapeutic intervention.

This study has several limitations. First, the signature has not been evaluated in large, prospective, multi-center independent cohorts, and its generalizability and clinical utility require further validation. Second, owing to heterogeneity in samples, sequencing technologies, and platforms across the datasets used in this study, batch effects in cross-modality analyses could not be fully eliminated. Third, our experimental validation remains limited. SRI has not been evaluated in vivo or in organoid systems, and its mechanisms of promoting gastric cancer remain undefined. The remaining signature genes also lack functional validation. These will be priorities for future work.

## 5. Conclusions

In summary, our multi-omics analysis uncovered a distinct malignant epithelial subset, C5, which shows broad enrichment of malignancy-related hallmark pathways and consistent associations with adverse prognosis across multiple cohorts. Intersection of C5 markers with tumor-specific upregulated genes yielded a five-gene prognostic signature that stratifies patients into high- and low-risk groups. These groups display divergent survival outcomes, immune infiltration characteristics, and immunotherapy responsiveness. Within the signature, TMEM176A and SRI were identified as risk genes associated with worse survival. Spatial transcriptomics localized TMEM176A primarily to fibroblasts and SRI to epithelial regions. In vitro functional assays demonstrated that SRI depletion hampers gastric cancer cell growth, migration, and invasion.

## Data Availability

All data used in this study are publicly available. Single-cell RNA-seq data were obtained from the Gene Expression Omnibus (GEO) (https://www.ncbi.nlm.nih.gov/geo/) (accessed on 20 May 2024) under accession GSE183904. Bulk RNA-seq data for gastric cancer were downloaded from The Cancer Genome Atlas via the Genomic Data Commons (https://portal.gdc.cancer.gov/) (accessed on 20 May 2024). Additional bulk transcriptomic validation cohorts were retrieved from GEO under accessions GSE15459, GSE26942 and GSE62254. Spatial transcriptomics data was accessed from GEO under accession GSE251950.

## Supplementary Materials

The following supporting information can be downloaded at: https://www.mdpi.com/article/10.3390/cancers17213483/s1, Figure S1: Single-cell RNA-seq QC. Figure S2: inferCNV-based copy number variation in epithelial cells. Figure S3: Associations of C3 infiltration with prognosis, epithelial cell differentiation trends, and risk–clinical correlations. Figure S4: Overall landscape of C5-mediated communication with the tumor microenvironment. Figure S5: Therapeutic response metrics by SRI expression. Table S1: Primer and siRNA sequences used in this study. Table S2: Marker genes for malignant epithelial cell subpopulations. Table S3: Significant intercellular signaling pathways among cell subpopulations. Table S4: Intersection of tumor-upregulated genes and C5 marker genes. Table S5: GO and KEGG enrichment analysis of C5-associated upregulated DEGs.

## Abbreviations

The following abbreviations are used in this manuscript: scRNA-seq: single-cell RNA sequencing; HER2:human epidermal growth factor receptor 2; PD-1:monoclonal antibodies targeting programmed cell death protein 1; GEO: Gene Expression Omnibus; TCGA: The Cancer Genome Atlas; VST: variance-stabilizing transformation; PCA: principal component analysis; UMAP: Uniform Manifold Approximation and Projection; TPM: transcripts per million; CNV: copy number variation; GSVA: gene set variation analysis; DEGs: differentially expressed genes; GO: Gene Ontology; KEGG: Kyoto Encyclopedia of Genes and Genomes; ROC: receiver operating characteristic; AUC: area under the curve; TME: tumor microenvironment; TIDE: Tumor Immune Dysfunction and Exclusion; IPS: immunophenoscore; TMB: tumor mutation burden; PPI: protein–protein interaction; NK cells: natural killer cells.
