# Supplementary material for: Integrated Multi-Omics Analysis Identifies SRI as a Critical Target Promoting Gastric Cancer Progression and Associated with Poor Prognosis
Source: Cancers (Basel). 2025 Oct 29;17(21):3483. doi: 10.3390/cancers17213483 (PMC12610080; doi:10.3390/cancers17213483)
Supplement: Supplementary file 1 [file cancers-17-03483-s001.zip › Supplementary_figures.pdf]

Supplementary Figures

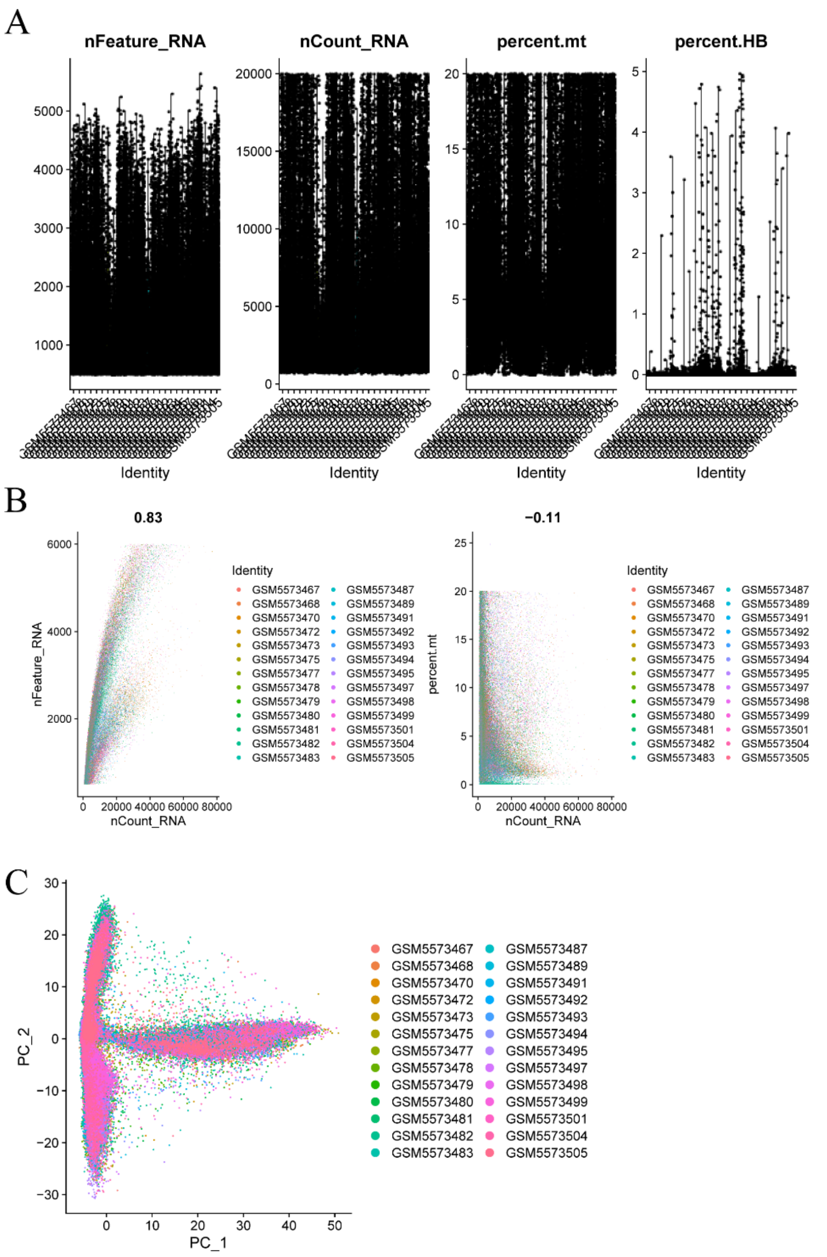

Figure S1. Single-cell RNA-seq QC. (**A**) Post-QC distributions of per-cell gene counts, UMI counts, mitochondrial % and hemoglobin % by sample. (**B**) nFeature_RNA vs. nCount_RNA scatter plots per sample with Pearson r. (**C**) PCA of QC metrics across samples.

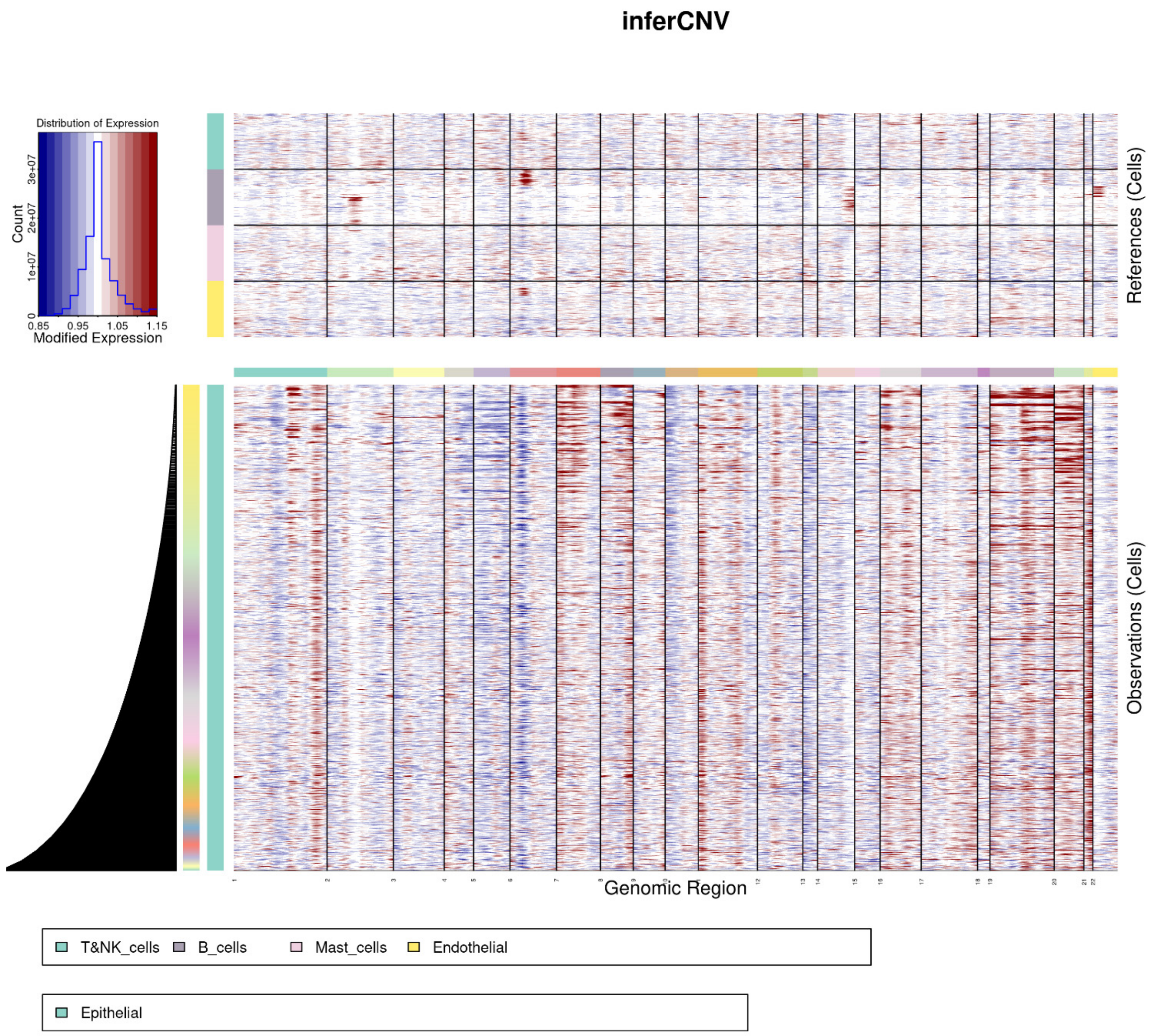

Figure S2. inferCNV-based copy number variation in epithelial cells.

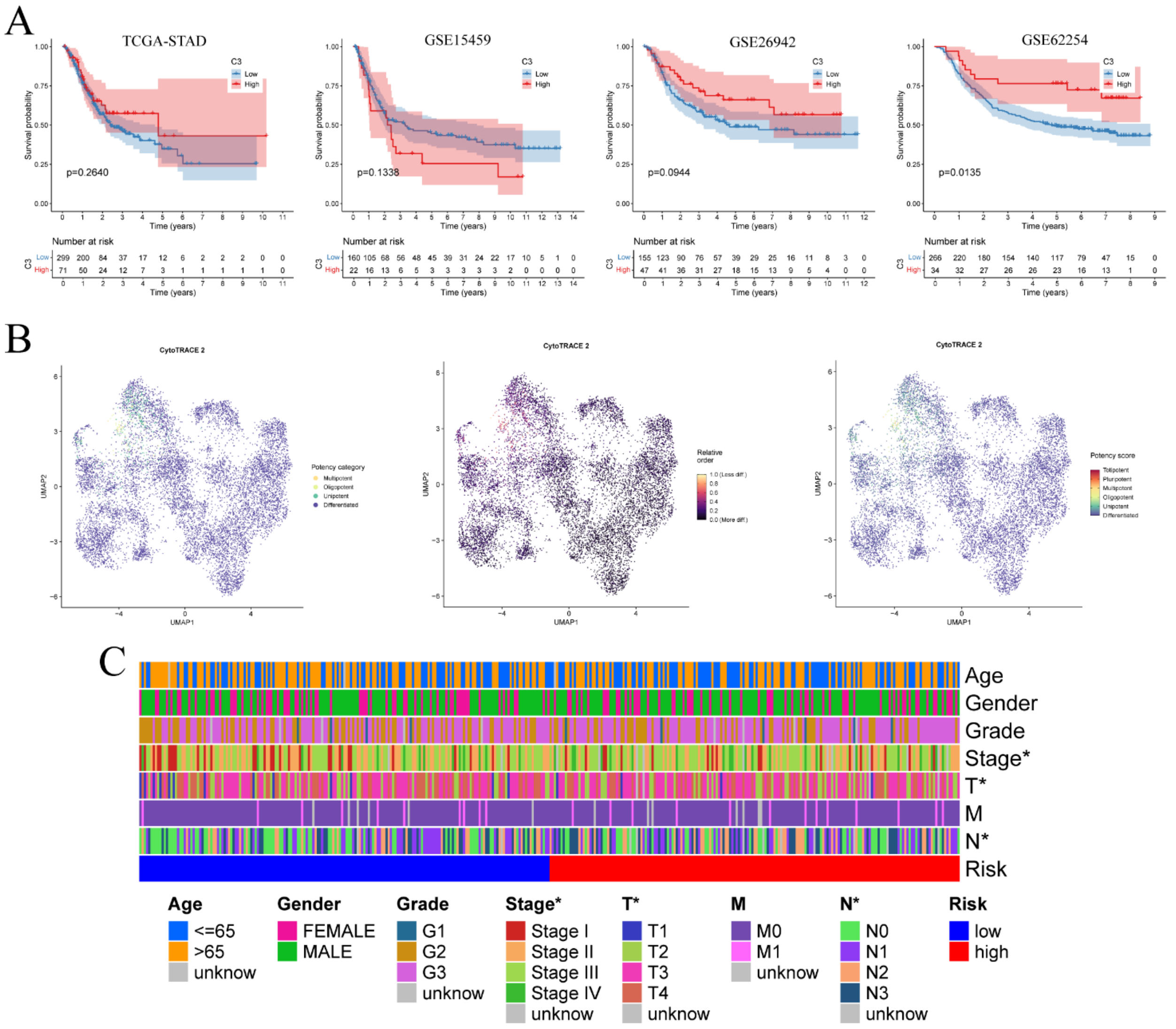

Figure S3. Associations of C3 infiltration with prognosis, epithelial cell differentiation trends, and risk–clinical correlations. (**A**) Kaplan–Meier analysis of overall survival stratified by low versus high C3 infiltration. (**B**) UMAP of CytoTRACE scores across molecular subtypes, indicating differentiation potential. (**C**) Correlation heatmap of risk groups with clinical parameters (age, gender, grade, T, N, M, stage). *$p < 0.05$, **$p < 0.01$, ***$p < 0.001$.

A

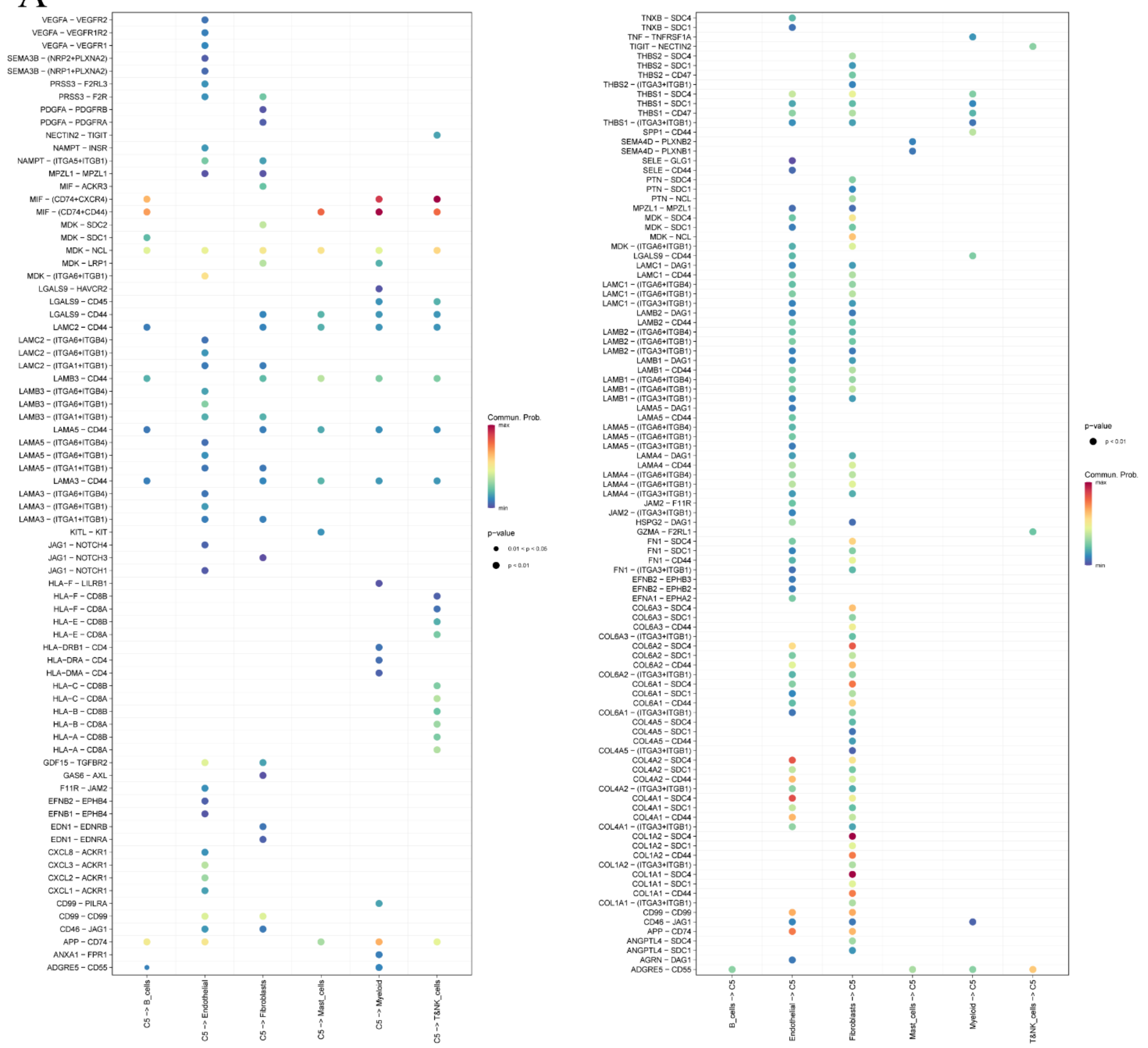

Figure S4. Overall landscape of C5-mediated communication with the tumor microenvironment.

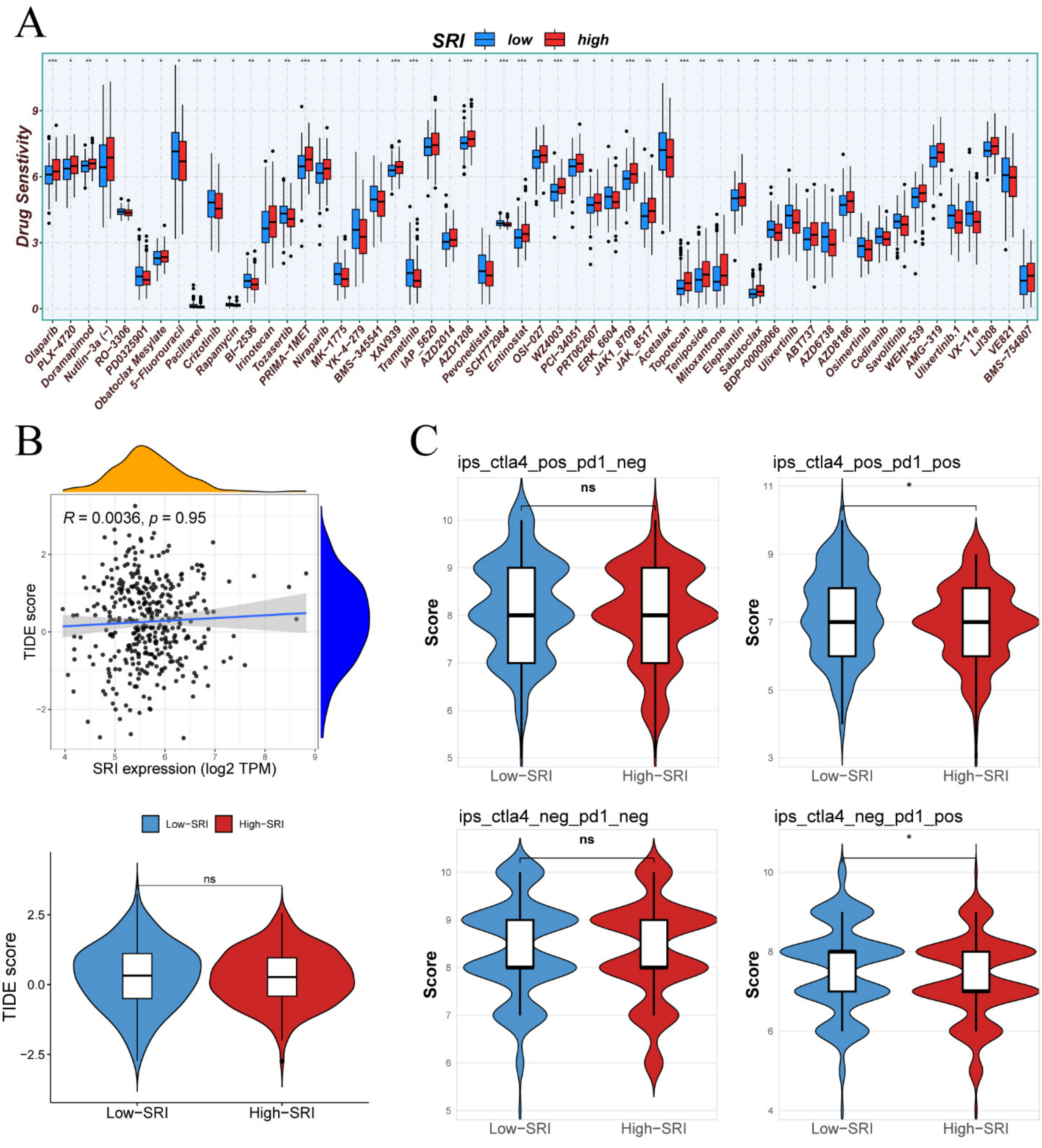

Figure S5. Therapeutic response metrics by SRI expression (**A**) Differential drug sensitivity between SRI-low and SRI-high groups. (**B**) TIDE score in relation to SRI expression and group. (**C**) IPS differences across CTLA4/PD1 subtypes by SRI group.
